# Alcohol use and its association with sexual risk behaviors in the Armed Forces of the Republic of the Congo

**DOI:** 10.1371/journal.pone.0223322

**Published:** 2019-10-02

**Authors:** Bonnie Robin Tran, Nicole Glass, Osika Tripathi, Olivier Kalombo, Pascal Ibata, Romain Bagamboula Mpassi

**Affiliations:** 1 Department of Defense HIV/AIDS Prevention Program, San Diego, California, United States of America; 2 Leidos Inc., Reston, Virginia, United States of America; 3 US Embassy, Kinshasa, Democratic Republic of the Congo; 4 Armed Forces of the Republic of the Congo, Brazzaville, Republic of the Congo; University of the Witwatersrand, SOUTH AFRICA

## Abstract

**Background:**

Previous research has shown alcohol misuse amplifies the risk of acquiring sexually transmitted infections [STIs], including HIV, by increasing high risk sexual behaviors. Military populations are particularly vulnerable to both alcohol misuse and STIs due to the unique conditions of military service. This study estimated the prevalence of probable hazardous and harmful alcohol use and examined associations with transactional sex, sex with a sex worker, and multiple sexual partners among military personnel in the Armed Forces of the Republic of the Congo (FAC).

**Methods:**

A secondary analysis of data collected from a 2014 seroprevalence and behavioral epidemiology risk survey was performed. Participants included 703 active duty male service members 18 years of age or older who reported ever having sex. Patterns of harmful and hazardous drinking were measured with the Alcohol Use Disorders Identification Test (AUDIT). Participants with an AUDIT score ≥ 8 (indicative of probable hazardous and harmful alcohol use, and possible alcohol dependence) were compared to those with an AUDIT score ≤ 7.

**Results:**

A total of 15.8% received a score of 8 or higher on the AUDIT. These participants were more likely to be lower educated and of lower military rank. In separate multivariable models, an AUDIT score ≥ 8 was significantly associated with higher odds of sex with a commercial sex worker and having multiple sexual partners.

**Conclusions:**

Study results emphasize the need to address patterns of harmful and hazardous alcohol use in the FAC and integrate alcohol misuse education into the HIV prevention program. The development of military-specific interventions to reduce alcohol-related risky sexual behaviors are also needed. Lastly, implementing policies such as restricting alcohol availability and sales on military bases, and adding warning labels to advertisements and containers may provide a more comprehensive response to reduce problematic alcohol use.

## Introduction

Hazardous and harmful alcohol use is a recognized public health problem in many countries around the world, with an estimated 3 million deaths attributed to dangerous use annually [1]. Unsafe alcohol use has both physical and psychological adverse effects and can cause long and short term damage to the health of those that consume harmful levels [1].

Previous systematic reviews and meta-analyses have shown unsafe alcohol use amplifies the risk of acquiring sexually transmitted infections, including HIV [2–6], by increasing high risk sexual behaviors [7]. Several theories have been proposed to explain the relation between alcohol consumption and sexual risk behaviors. The alcohol myopia theory asserts that alcohol consumption restricts cognitive capacity and causes people to focus on the most salient cues (e.g., fulfilling sexual pleasure) instead of more abstract or distal cues (e.g., a partner’s level of disease risk) [8]. Conversely, the alcohol expectancy theory proposes that a person’s belief about the effects of alcohol consumption may influence subsequent behavior. For example, those who believe that alcohol enhances sexual arousal and performance are more likely to have sex under the influence of alcohol [9]. Other researchers have proposed that personality traits such as sensation seeking or compulsivity may help explain the association between alcohol use and sexual risk behavior [10].

Investigating alcohol use in relation to risky sexual behaviors in sub-Saharan Africa is of particular importance. In recent years, the African continent has been identified as a growth market for alcoholic beverage companies due to increased economic development and urbanization. Beer sales are projected to grow at a faster rate in Africa compared to other regions in the world [11]. This is a cause for concern, especially in the context of the HIV epidemic: sub-Saharan Africa accounts for nearly 70% of all people living with HIV [12]; hazardous and harmful alcohol use can further perpetuate the HIV epidemic by increasing risky sexual behaviors.

Military populations may be particularly vulnerable to both hazardous and harmful alcohol consumption and HIV infection due to the unique conditions of military service. Previous research in sub-Saharan African militaries has found that a higher proportion of service members exhibited patterns of excessive alcohol consumption compared to the general population [13, 14]. Quantitative and qualitative studies have shown that military personnel may be more likely to abuse alcohol to manage post-combat stress or trauma [15], cope with frequent and lengthy deployments and work-related stress, alleviate boredom, or deal with uncertainties of military service (e.g., long waiting periods to learn about new assignments, life-threatening deployments, and other life-changing transitions which may impede a soldier’s ability to plan for the future) [16, 17]. Studies have also shown HIV prevalence to be higher among some sub-Saharan African military populations compared to their civilian counterparts [18, 19]. Furthermore, prior research has linked unsafe alcohol use to risky sexual behaviors among service members in sub-Saharan Africa [14, 20]. For example, a study of the Angolan Armed Forces reported that alcohol use before sex and problematic drinking were predictive of a higher number of sexual partners [20]. Another study in the Malawi Defence Force showed elevated odds of participation in transactional sex and having multiple sexual partners among male service members showing patterns of problematic alcohol use [14]. While these studies add to generalizable knowledge regarding the associations of alcohol misuse with sexual risk behaviors, further studies in other sub-Saharan African militaries are needed to elucidate this relation and to develop appropriate military-specific interventions.

The objectives of the present study were to 1) estimate the prevalence of probable hazardous and harmful alcohol use, 2) identify associated factors, and 3) examine associations of probable hazardous and harmful alcohol use with risky sexual behaviors among military personnel in the Armed Forces of the Republic of the Congo (FAC). This study aimed to add to the paucity of research on alcohol misuse and associations with sexual risk behaviors among sub-Saharan African militaries.

## Methods

### Study design and participants

This study was a secondary analysis of data collected from a seroprevalence and behavioral epidemiology risk survey (SABERS) [21] in the FAC. The FAC SABERS estimated the prevalence of HIV and syphilis, and identified associated factors. The FAC SABERS was a cross-sectional study conducted from April to May 2014 at military bases that were proportionally representative of the overall FAC. Military units at the bases were randomly selected for inclusion in the study. Eligible participants from these units included active duty male and female service members who were 18 years of age or older. Due to the small proportion of female service members and those with a ranking of officer, all individuals who met these characteristics from the selected military units were invited to an informational briefing to increase representation. Lower ranked male service members were selected using a random process. Those who drew a piece of paper from a hat with an “X” marked on it were invited to the informational briefing where the study purpose and procedures were explained. All individuals were able to ask study personnel questions about their participation.

The FAC SABERS was conducted in compliance with all applicable federal regulations governing the protection of human subjects in research and approved by institutional review boards in the US (Naval Health Research Center [NHRC], San Diego, California; Protocol No.: NHRC.2013.0021) and in the Republic of the Congo (ROC) (Ministry of Scientific Research and Technological Innovation, Brazzaville, ROC; No. 00000230/DGRST/CERSSA). Human subjects participated in the FAC SABERS after providing electronic informed consent. Study materials (e.g., consent form, survey) were developed in English and translated to French, the national language of ROC, by a certified translation company (Lingua Consult, Yaoundé, Cameroon).

### Study procedures

Consented participants completed an anonymous survey through a computer-assisted personal interview facilitated by trained study personnel. No personally identifiable information (e.g., name, date of birth, military or national identification numbers) was collected. Interviews were conducted in a private setting. Participants were instructed they could skip any questions they did not feel comfortable answering.

### Variables and measures

Variables of interest included demographics, military characteristics, mental health, alcohol use, and sexual history. Demographics consisted of age, highest educational attainment (did not attend or complete primary school, completed primary school, completed secondary school, completed high school or higher), and marital status (married [included monogamous and polygamous marriage], not married but living with a partner, never married and not living with a partner, widowed/divorced/separated). Military characteristics included military rank (soldier/corporal, non-commissioned officer, officer), military branch (Army, Navy, Air Force, Gendarmerie), years of military service (0–4, 5–10, 11+) and local and foreign deployments in the last 6 months (no, yes).

Mental health problems of interest included post-traumatic stress disorder (PTSD) and depression. PTSD was assessed using the 4-item primary care post-traumatic stress disorder (PC-PTSD) screen, a validated tool developed to identify probable PTSD among US military veterans [22]. This tool has been used among civilians in sub-Saharan Africa [23]. An affirmative response to an item was given a score of 1 and all items were summed for a final score that ranged from 0 to 4. Participants with a score of 3 or higher were classified as positive for probable PTSD. Depression was assessed using the Patient Health Questionnaire-9 (PHQ-9), a validated screening tool for the detection of depression and severity based on the Fourth Edition of the Diagnostic and Statistical Manual of Mental Disorders (DSM-IV) [24]. This tool has also been validated in sub-Saharan African civilian populations [25, 26] and used in US military populations [27, 28]. Each item was scored from 0 to 3 and summed to create a final score that ranged from 0 to 27. A score of 0 indicated no depression, 1 to 4 minimal depression, 5 to 9 mild depression, 10 to 14 moderate depression, 15 to 19 moderately severe depression, and 20 or higher severe depression. Due to small numbers, those with a score of 5 or higher were collapsed into 1 group (i.e., those exhibiting mild or more severe depression).

Hazardous and harmful alcohol use, and possible alcohol dependence, was measured with the Alcohol Use Disorders Identification Test (AUDIT), a screening tool developed by the World Health Organization [29]. This tool has been validated among men and women and previously used in other African military populations [13, 14, 20, 30]. The AUDIT has also been translated to many different languages; the French version was used in the FAC SABERS. The AUDIT consisted of 10 questions, which were scored on a scale that ranged from 0 to 4. Responses to each question were summed, for a total score that varied from 0 to 40. The total AUDIT score reflected a participant’s level of risk related to alcohol. A score of 0 was an indicator of no alcohol use, 1 to 7 an indicator of low risk alcohol use, and 8 or higher an indicator of probable hazardous and harmful alcohol use, and possible alcohol dependence. Participants with a score of 8 or higher were compared to those with a score of 7 or lower. Additional questions were asked about condom use and unintentional sex under the influence of alcohol in the last 3 months (no, yes). These questions were “In the last three months, did drinking alcohol influence your decision about or prevent you from using condoms or using condoms correctly?” and “In the last three months, did you ever have unintended sex, as a result of drinking alcohol?”.

History of risky sexual behaviors included sex with a commercial sex worker (CSW), multiple sexual partners, and transactional sex. Participants were asked the number of CSWs they had sex with in the past 12 months. Those who responded with a number of 1 or higher were classified as having engaged in sex with a CSW and were compared to those that had not. Participants were asked if they had more than 1 sexual partner in the same week (no, yes). Lastly, participants were asked whether they had ever participated in transactional sex (i.e., ever paid or received money, shelter, food, drugs, favors, or gifts in exchange for sex). Those who responded they had paid or received money or goods in exchange for sex were categorized into 1 group due to small numbers and compared with participants who had never participated in transactional sex. No participants in this study reported both having paid and received money or goods in exchange for sex.

### Statistical analysis

Descriptive statistics were performed for all variables and associations with probable hazardous and harmful alcohol use were estimated using bivariate logistic regressions. The associations of probable hazardous and harmful alcohol use with risky sexual behaviors (sex with a CSW, multiple sexual partners, and transactional sex) were independently assessed using multivariable logistic regressions. Each multivariable model was adjusted using *a priori* identified confounding variables in the causal pathway: age, education, marital status, and military rank. This method for identification of confounders has been previously described [31]. All models were assessed for possible collinearity by observing tolerance; values <0.10 indicated collinearity. Odds ratios and 95% confidence intervals were estimated for all logistic regression models and statistical significance of variable effects was determined using the Wald chi-square value with an alpha of 0.05. All analyses were conducted using SAS software, version 9.4 (SAS Institute, Inc., Cary, NC).

## Results

**Fig 1:**
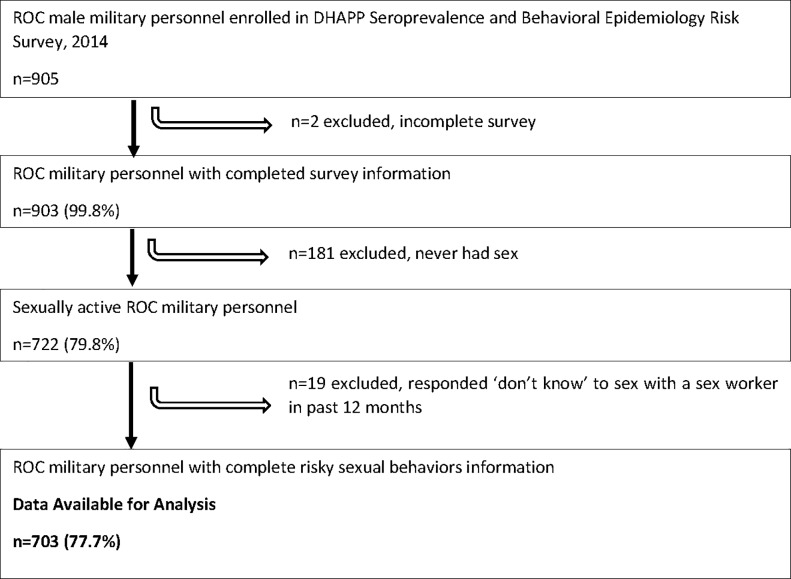
Strobe diagram for identifying associations between probable hazardous and harmful alcohol use and risky sexual behaviors among male military personnel in the Armed Forces of the Republic of the Congo, 2014.

A total of 982 FAC military personnel were enrolled in the SABERS study. Due to the small number of women who consented (n = 77), analyses were restricted to 905 men. Of these men, 202 were excluded from the current analysis for the following reasons: 2 had incomplete survey information, 181 reported no lifetime sexual activity, and 19 provided a response of ‘don’t know’ to sex with a sex worker in the past 12 months (Fig 1). This resulted in a final sample of 703 for secondary analyses. Sensitivity analyses were performed to assess differences in demographics, military characteristics, mental health, and alcohol use between participants who were excluded and those who were included in the analysis. Those who were excluded had lower levels of education, were unmarried (either not living with a partner or living with a partner), were less likely to have been on a local deployment, but more likely to have been on a foreign deployment, and less likely to have screened positive for probable PTSD or depression. Alcohol use did not differ significantly between the two groups.

The mean age was 37.2 years (standard deviation = 7.0; range = 23–60; Table 1). The majority of participants were not married but were living with a partner (67.1%) and had less than a secondary school education (52.6%). The Army represented the largest proportion from the four military branches (81.2%). Most of the participants were ranked as a non-commissioned officer (57.6%) and had served in the military for 11 or more years (79.0%). A higher percentage of participants reported a local deployment (16.9%) than a foreign deployment (7.8%). A total of 8.8% of participants screened positive for probable PTSD and 7.2% screened positive for mild or more severe depression.

**Table 1:** Description of sexually active male military personnel in the Armed Forces of the Republic of the Congo (n = 703).

Characteristic	n	%
**Age**		
Mean (standard deviation)	37.2	(7.0)
Median (range)	36	(23–60)
**Education**		
Completed high school or higher	156	22.2
Completed secondary school	177	25.2
Completed primary school	191	27.2
Did not attend or complete primary school	179	25.4
**Marital Status**		
Married (monogamous or polygamous)	209	29.7
Not married but living with a partner	472	67.1
Never married and not living with a partner	16	2.3
Widowed/divorced/separated	6	0.9
**Military Branch**		
Army	571	81.2
Navy	35	5.0
Air Force	19	2.7
Gendarmerie	78	11.1
**Military Rank**		
Soldier/corporal	155	22.1
Non-commissioned officer	405	57.6
Officer	143	20.3
**Years of Military Service**		
0–4 years	35	5.0
5–10 years	113	16.0
11+ years	555	79.0
**Local Deployment**		
No	584	83.1
Yes	119	16.9
**Foreign Deployment**		
No	648	92.2
Yes	55	7.8
**PC-PTSD Screening Tool**		
Negative for probable PTSD	641	91.2
Positive for probable PTSD	62	8.8
**PHQ-9 Depression Screening Tool**		
No depression	570	81.1
Minimal depression	82	11.7
Mild or more severe depression	51	7.2

A majority (77.2%) of the participants had consumed alcohol in the past year (Table 2). Of all participants, 15.8% had a score of 8 or higher on the AUDIT. Over a third (37.6%) of participants reported that in the last three months alcohol use had influenced their decision to use condoms or use them correctly and over one in five (21.9%) reported they had unintended sex as a result of alcohol use.

**Table 2:** Prevalence of probable hazardous and harmful alcohol use and influence on sexual behaviors among sexually active male military personnel in the Armed Forces of the Republic of the Congo (n = 703).

	n	%
**AUDIT Score**		
0: no alcohol use	160	22.8
1 to 7: low risk alcohol use	432	61.4
8 or higher: probable harmful and hazardous alcohol use, and possible alcohol dependence	111	15.8
**Incorrect condom use as a result of drinking**	264	37.6
**Unintended sex as a result of drinking**	154	21.9

Educational attainment and military rank were independently associated with an AUDIT score of 8 or higher (Table 3). The odds of scoring 8 or higher on the AUDIT were higher among those who did not attend or complete primary school (odds ratio [OR] 1.99, 95% confidence interval [CI] 1.06–3.71) and among those who completed primary school (OR 1.96, 95% CI 1.06–3.65) compared to those who completed high school or higher. The odds of scoring 8 or higher on the AUDIT were also elevated among participants of lower rank (soldier/corporal) compared to officers (OR 2.59, 95% CI 1.33–5.06). Lower age, the Navy and Air Force branch, and probable PTSD showed marginal associations with an AUDIT score of 8 or higher (p = 0.07, p = 0.06, and p = 0.07, respectively).

**Table 3:** Factors associated with probable hazardous and harmful alcohol use among sexually active male military personnel in the Armed Forces of the Republic of the Congo (n = 703).

	No/Low Risk Alcohol Use		Probable Hazardous and Harmful Alcohol Use				
	(n = 592)		(n = 111)				
**Characteristic**	**n**	**%**	**n**	**%**	**OR**	**95% CI**	**p-value**
**Age**							0.07
Mean (standard deviation)	37.4	(7.2)	36.1	(6.0)	0.97	0.94–1.00	
Median (range)	37	(23–60)	36	(26–53)			
**Education**							0.04
Completed high school or higher	139	23.5	17	15.3	1.00	(ref)	
Completed secondary school	155	26.2	22	19.8	1.16	0.59–2.28	
Completed primary school	154	26.0	37	33.4	1.96	1.06–3.65	
Did not attend or complete primary school	144	24.3	35	31.5	1.99	1.06–3.71	
**Marital Status**							0.23
Married (monogamous or polygamous)	169	28.5	40	36.0	1.00	(ref)	
Not married, living with a partner	406	68.6	66	59.5	0.69	0.45–1.06	
Never married, not living with a partner	13	2.2	3	2.7	0.98	0.27–3.58	
Widowed/divorced/separated	4	0.7	2	1.8	2.11	0.37–11.9	
**Military Branch**							0.06
Army	483	81.6	88	79.3	1.00	(ref)	
Navy	25	4.2	10	9.0	2.20	1.02–4.73	
Air Force	14	2.4	5	4.5	1.96	0.69–5.58	
Gendarmerie	70	11.8	8	7.2	0.63	0.29–1.35	
**Military Rank**							0.02
Officer	129	21.8	14	12.6	1.00	(ref)	
Non-commissioned officer	342	57.8	63	56.8	1.70	0.92–3.14	
Soldier/corporal	121	20.4	34	30.6	2.59	1.33–5.06	
**Years of Military Service**							0.44
0–4 years	32	5.4	3	2.7	1.00	(ref)	
5–10 years	93	15.7	20	18.0	2.29	0.64–8.24	
11+ years	467	78.9	88	79.3	2.01	0.60–6.71	
**Local Deployment**							0.83
No	491	82.9	93	83.8	1.00	(ref)	
Yes	101	17.1	18	16.2	0.94	0.54–1.63	
**Foreign Deployment**							0.52
No	544	91.9	104	93.7	1.00	(ref)	
Yes	48	8.1	7	6.3	0.76	0.34–1.73	
**PC-PTSD Screening Tool**							0.06
Negative for probable PTSD	545	92.1	96	86.5	1.00	(ref)	
Positive for probable PTSD	47	7.9	15	13.5	1.81	0.97–3.37	
**PHQ-9 Depression Screening Tool**							0.27
No depression	485	81.9	85	76.6	1.00	(ref)	
Minimal depression	64	10.8	18	16.2	1.61	0.91–2.84	
Mild or more severe depression	43	7.3	8	7.2	1.06	0.48–2.34	

OR: odds ratio; CI: confidence interval

In the final multivariable models, after controlling for age, education, marital status, and military rank, an AUDIT score of 8 or higher was significantly associated with higher odds of sex with a CSW (adjusted odds ratio [aOR] 2.43, 95% CI 1.15–5.16) and having multiple sexual partners (aOR 1.76, 95% CI 1.09–2.82) (Table 4). No association with transactional sex was observed (aOR 0.82, 95% CI 0.49–1.36). Collinearity was absent from all models.

**Table 4:** Multivariable models of probable hazardous and harmful alcohol use and risky sexual behaviors among male military personnel in the Armed Forces of the Republic of the Congo (n = 703).

	Probable Hazardous and Harmful Alcohol Use		
Outcome ^a^	aOR	95% CI	p-value
Sex with a commercial sex worker	2.43	(1.15–5.16)	0.02
Multiple sexual partners	1.76	(1.09–2.82)	0.02
Participation in transactional sex ^b^	0.82	(0.49–1.36)	0.44

aOR: adjusted odds ratio; CI: confidence interval

^a^ Adjusted for age, education, marital status, military rank

^b^ Paid or received money or goods for sex

## Discussion

This study determined several correlates of probable hazardous and harmful alcohol use (and possible alcohol dependence) and identified risky sexual behaviors associated with adverse alcohol consumption among the Armed Forces of the Republic of the Congo (FAC). To our knowledge, no prior research on alcohol use and sexual risk behaviors among FAC military personnel has been conducted.

Alcohol consumption was relatively common among the FAC, with a majority (77.2%) of participants reporting consuming alcohol in the past year. Of concern is that 15.8% of all participants met criteria indicative of hazardous and harmful alcohol use and possible alcohol dependence (i.e., AUDIT score ≥ 8). Furthermore, this estimate is over 4 times higher than the estimate reported among men in the general population (3.8%) [32]. These findings align with previous research among military personnel demonstrating a higher prevalence of alcohol use disorders among service members compared to civilians [13, 14]. Studies have shown that the unique challenges of military service including exposure to combat, trauma, frequent and lengthy deployments, and uncertainties in military service (e.g., next assignment) may propel service members to turn to alcohol to manage these stressors [15–17]. Additionally, research has suggested that the endorsement of gender norms perpetuating masculinity, physical strength, and mental toughness may prevent service members from seeking treatment for medical conditions [33], and compel them to turn to alcohol to deal with their problems. Given the unique structure of military systems, integrating alcohol abuse prevention, screening, and treatment into a soldiers’ routine health assessment may help improve and preserve force health and readiness. Additionally, limiting the availability of alcoholic beverages on the base, increasing taxes on sales, or requiring health warning labels on advertisements or containers (policies on this are currently lacking in ROC [32]) may help reduce alcoholic consumption [34]. Other prevention measures that promote physical activity have also been shown to have promising outcomes (e.g., improved abstinence and reduced alcohol consumption) for those with an alcohol use disorder, although further research on exercise interventions is needed [35].

An AUDIT score of 8 or higher was found to be associated with lower military rank. These findings align with previous research in other countries that found rates of heavy drinking were substantially higher among lower-ranked military personnel [36, 37]. The culture of heavy drinking may be more common among the lower ranks to cope with peer pressure and aspects of military service, such as deployments to remote locations for lengthy periods of time. Recruits into the FAC should be screened using the AUDIT, and those identified as at-risk for probable alcohol misuse (score of 8 or higher) should not be allowed to join the FAC. This study also found that lower education was linked to an AUDIT score of 8 or higher. These findings suggest that FAC members may benefit from educational messages on the negative effects of harmful, hazardous, and dependent alcohol use and the role alcohol use may play in HIV infection and transmission. While these messages would help all military members, understanding other factors that may influence alcohol misuse among those with lower education levels is important to develop proper interventions.

While the association between alcohol use and PTSD was not statistically significant, the trend (i.e., elevated odds of 1.81) that was observed in this study merits further investigation. A sizeable proportion of participants (8.8%) screened positive for probable PTSD. Studies in service members have shown alcohol use to be highly correlated with PTSD [38, 39]. Those suffering from PTSD may be more likely to abuse alcohol to handle painful and stressful memories associated with experiences of traumatic events. Co-occurring PTSD and alcohol misuse can lead to detrimental physical, psychological, and adverse sexual health outcomes, which ultimately can impact force readiness. Additional studies are needed to examine these associations further in FAC service members.

With regards to associations of alcohol use with risky sexual behaviors, an AUDIT score of 8 or higher was linked to higher odds of sex with a CSW. Engaging in sex with a sex worker can increase one’s exposure to HIV. A systematic review and meta-analysis reported that the burden of HIV disease is disproportionately higher among female sex workers (FSWs) [40]. Furthermore, research has suggested that male military members regularly engage in sex with FSWs and often do not practice safe sexual behaviors (including low condom use) with these partners [20, 41, 42]. An AUDIT score of 8 or higher was also shown to be associated with multiple sexual partners, as reported in other military populations [14, 20]. While we were unable to discern whether these partnerships were serial or concurrent, studies suggest that having multiple sexual partners, especially those that are concurrent, may be associated with higher risk of HIV acquisition and transmission [43, 44]. These findings add to growing evidence that alcohol use and HIV are very much intertwined public health issues among military personnel and should be addressed conjointly and comprehensively in HIV prevention programs.

While the previous results provide evidence of global associations between alcohol use and risky sexual behaviors, our study findings also suggest alcohol use may have a direct effect on one’s sexual behaviors. A substantial percentage of participants, regardless of their AUDIT score, reported that alcohol use led to unintentional sex (21.9%) or influenced their decision to use condoms or the ability to use them correctly (37.6%). These findings indicate that alcohol use can influence one’s ability to make decisions regarding safe sexual practices and educational messages should target all individuals who are sexually active and consuming alcohol. Additional information on the quantity of alcohol consumed, intoxication levels, which partner was consuming alcohol, and how often these behaviors occurred as a result of alcohol consumption may help researchers examine the effects of alcohol at specific levels of intoxication, gender differences, and the frequency of the behavior, as demonstrated in other research [45].

There were several limitations to this study. Due to concern with low literacy rates in the FAC, all data were collected through a personal interview, which may have increased social-desirability bias. However, participants were told they could refuse to answer any questions they did not feel comfortable providing a response to. Interview bias was also plausible. To minimize this, all study interviewers were trained on ethical and proper data collection techniques. Comparisons of the prevalence of probable hazardous and harmful alcohol use in service members vs. civilians should be interpreted with some limitation in mind as different measurements may have been used (i.e., the AUDIT was used in the current analysis; it is unclear what measurement was used in the WHO global report on alcohol use [32]). Causality and temporality cannot be determined due to the cross-sectional study design. Additionally, results of the sensitivity analysis found differences in demographic, military, and mental health characteristics for participants who were excluded from the analysis–therefore results should be interpreted with caution. Due to the use of convenience sampling for those with a ranking of officer, this group was over-represented and selection bias may have been introduced in the FAC SABERS. Due to the small number of female participants included in the original study, analyses on women were not performed and these results are therefore not generalizable to female FAC service members. Further studies among a large sample of female service members are needed to elucidate the relation between alcohol use and risky sexual behaviors.

In conclusion, the role of unsafe alcohol consumption in the acquisition and transmission of HIV, through risky sexual behaviors, among military populations in sub-Saharan Africa is concerning. Integrating alcohol abuse prevention, screening, and care and treatment into existing HIV prevention programs is essential to maintain a healthy and effective military. The development of military-specific interventions to reduce alcohol-related risky sexual behaviors are also needed, taking into account the idiosyncrasies of military service. Lastly, increasing the tax on alcohol purchases, as well as adding warning labels to advertisements and containers may help provide a more comprehensive response to reduce problematic alcohol use.
